# Realization of Spin‐locked Acoustic Helical Landau Levels in both Hexagonal and Square Lattices

**DOI:** 10.1002/advs.202507059

**Published:** 2025-06-27

**Authors:** Yafeng Chen, Zhihao Lan, Shanjun Liang, Lei Fan, Jie Zhu, Zhongqing Su

**Affiliations:** ^1^ Institute of Acoustics School of Physics Science and Engineering Tongji University Shanghai 200092 China; ^2^ Department of Electronic and Electrical Engineering University College London London WC1E 7JE UK; ^3^ Division of Science, Engineering and Health Studies College of Professional and Continuing Education Hong Kong Polytechnic University Kowloon Hong Kong SAR 999077 China; ^4^ Department of Mechanical Engineering The Hong Kong Polytechnic University Kowloon Hong Kong SAR 999077 China

**Keywords:** helical landau levels, in‐plane pseudomagnetic fields, large‐area acoustic energy transportation

## Abstract

Topological zeroth‐order Landau levels offer a promising avenue for steering acoustic and electromagnetic waves. However, the helical Landau levels in 2D acoustic systems remain an unresolved challenge. Moreover, previous studies on zeroth‐order Landau levels have primarily focused on hexagonal lattices, leaving their counterparts in square lattices largely unexplored. In this study, the helical Landau levels rooted in acoustic quantum spin Hall systems are theoretically proposed and experimentally validated. By linearly increasing the local bandgap through the lifting of the double Dirac cone in acoustic crystals with both square and hexagonal lattices—achieved via topology optimization method—a position‐dependent effective mass is introduced in the Dirac Hamiltonian, thereby synthesizing in‐plane pseudomagnetic fields. This results in the emergence of spin‐locked helical Landau levels, which has been experimentally validated. The large‐area conveyance of acoustic energy facilitated by these helical Landau levels is demonstrated and the robustness of Landau‐level‐mediated propagation against defects is confirmed. A new pathway for exploring acoustic helical Landau levels based on spin Hall physics is opened.

## Graphical abstract

Acoustic helical Landau levels rooted in quantum spin Hall systems are achieved by linearly increasing the local bandgap through the lifting of the double Dirac cone in acoustic crystals. Large‐area conveyance of acoustic energy facilitated by these helical Landau levels is demonstrated. The designed structure offers a platform for exploring acoustic helical Landau levels based on spin Hall physics.

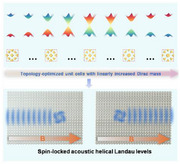

## 1 Introduction

In condensed matter physics, Landau quantization, a concept named after physicist Lev Landau, describes how the cyclotron orbits of charged particles become quantized when subjected to a uniform magnetic field. This intriguing phenomenon results in the formation of discrete energy levels, which are referred to as Landau levels, as well as quantum Hall effects.^[^ ^1^, ^2^ ^]^ Recently, by mimicking the mechanism of Landau quantization, Landau levels have been achieved in acoustic systems via constructing the strain‐induced pseudomagnetic fields.^[^ ^3^, ^4^ ^]^ Such pseudomagnetic fields are mainly synthesized by shifting the Dirac points within the 2D momentum space, which are out‐of‐plane and the induced Landau bands are flat and featured with a high density of states.^[^ ^5^, ^6^ ^]^ However, the waves associated with these flat bands are localized, preventing their propagation through the system.

Apart from flat Landau bands, recent studies demonstrated that dispersive chiral zeroth‐order Landau levels could be formed in 3D Weyl semimetals when subjected to external magnetic fields.^[^ ^7^, ^8^, ^9^, ^10^, ^11^ ^]^ These chiral Landau levels are topologically protected one‐way bulk states, with their propagation direction governed by the orientation of the external magnetic field and the net number of such levels determined by the topological charge of the degenerate point.^[^ ^12^ ^]^ Such chiral Landau levels have been realized in 3D acoustic and photonic Weyl metamaterial systems.^[^ ^13^, ^14^, ^15^, ^16^, ^17^ ^]^ Compared with 3D Weyl systems with complicated configurations, their 2D counterparts are more convenient for fabrication, making them highly promising for various applications. Recently, experimental demonstrations of chiral Landau levels, realized based on quantum valley Hall physics, have been achieved in 2D photonic and acoustic systems by synthesizing in‐plane pseudomagnetic fields.^[^ ^18^, ^19^, ^20^, ^21^, ^22^, ^23^, ^24^, ^25^ ^]^ Meanwhile, using the synthetic dimension methodology, chiral Landau levels in 2D valley sonic crystals are also achieved.^[^ ^26^ ^]^ Moreover, compared with the chiral zeroth‐order Landau levels in 3D Weyl semimetals, 3D Dirac semimetals that host two degenerate Weyl nodes with opposite chiralities at the same location in momentum space can support spin‐locked zeroth‐order Landau levels.^[^ ^27^, ^28^, ^29^ ^]^ However, in current studies on zeroth‐order Landau levels in 2D classical wave systems, the synthetic in‐plane pseudomagnetic fields are mainly constructed based on the valley‐Hall systems mimicking the two‐fold degenerate Weyl points. In 2D systems, apart from the valley Hall physics,^[^ ^30^, ^31^, ^32^ ^]^ the spin Hall physics relying on four‐fold degenerate Dirac points has also aroused great attention.^[^ ^33^, ^34^, ^35^, ^36^, ^37^, ^38^, ^39^, ^40^, ^41^ ^]^ However, helical acoustic zeroth‐order Landau levels based on spin Hall physics have not been reported yet. Besides, current 2D photonic and acoustic systems that host topological zeroth‐order Landau levels are mainly constructed with unit cells in hexagonal lattices. As lattice symmetry plays a vital role in topological systems, it is highly desirable to investigate systems that host topological zeroth‐order Landau levels beyond hexagonal lattices, such as square lattices.

In this work, we theoretically propose and experimentally demonstrate spin‐locked helical zeroth‐order Landau levels in 2D acoustic systems with both square and hexagonal lattices. In particular, we synthesize in‐plane pseudomagnetic fields in 2D inhomogeneous acoustic systems by strategically engineering a position‐dependent effective mass in the Dirac Hamiltonian via linearly opening the local bandgap of the double Dirac cone enabled by the topology optimization method.^[^ ^42^ ^]^ In doing so, the spin‐locked helical Landau levels are induced. We measure the band diagrams of the zeroth‐order Landau levels in experiments, which show decent agreements with the theoretical results. Meanwhile, we also experimentally validate the robustness of the Landau‐level‐mediated propagation against defects. Our work opens the door to investigate acoustic helical Landau‐levels based on spin Hall physics.

## 2 Results

**Figure 1:**
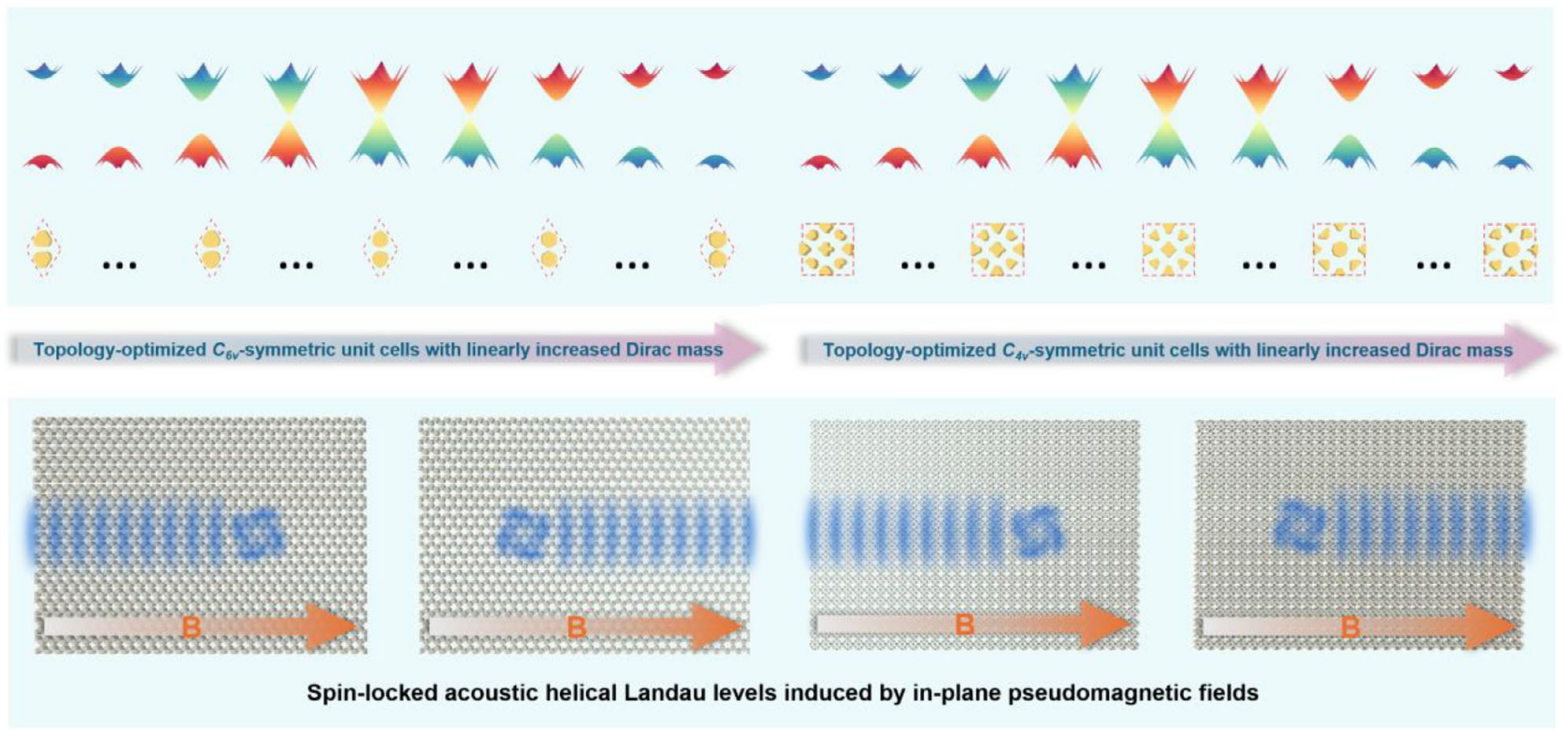
Schematic of the emergence of spin‐locked acoustic helical Landau levels.

Different from previous works that achieve chiral Landau levels in 2D acoustic or photonic systems based on quantum valley Hall physics, we herein engineer acoustic helical Landau levels based on quantum spin Hall physics in both hexagonal and square lattices, for which the conceptual schematic is illustrated in **Figure** **1**. Both *C_6v_*‐ and *C_4v_*‐symmetric unit cells with linearly increased effective Dirac mass are generated via topology optimization technique, the assembly of which induces in‐plane synthetic pseudomagnetic fields, leading to the emergence of spin‐locked acoustic helical Landau levels.

**Figure 2:**
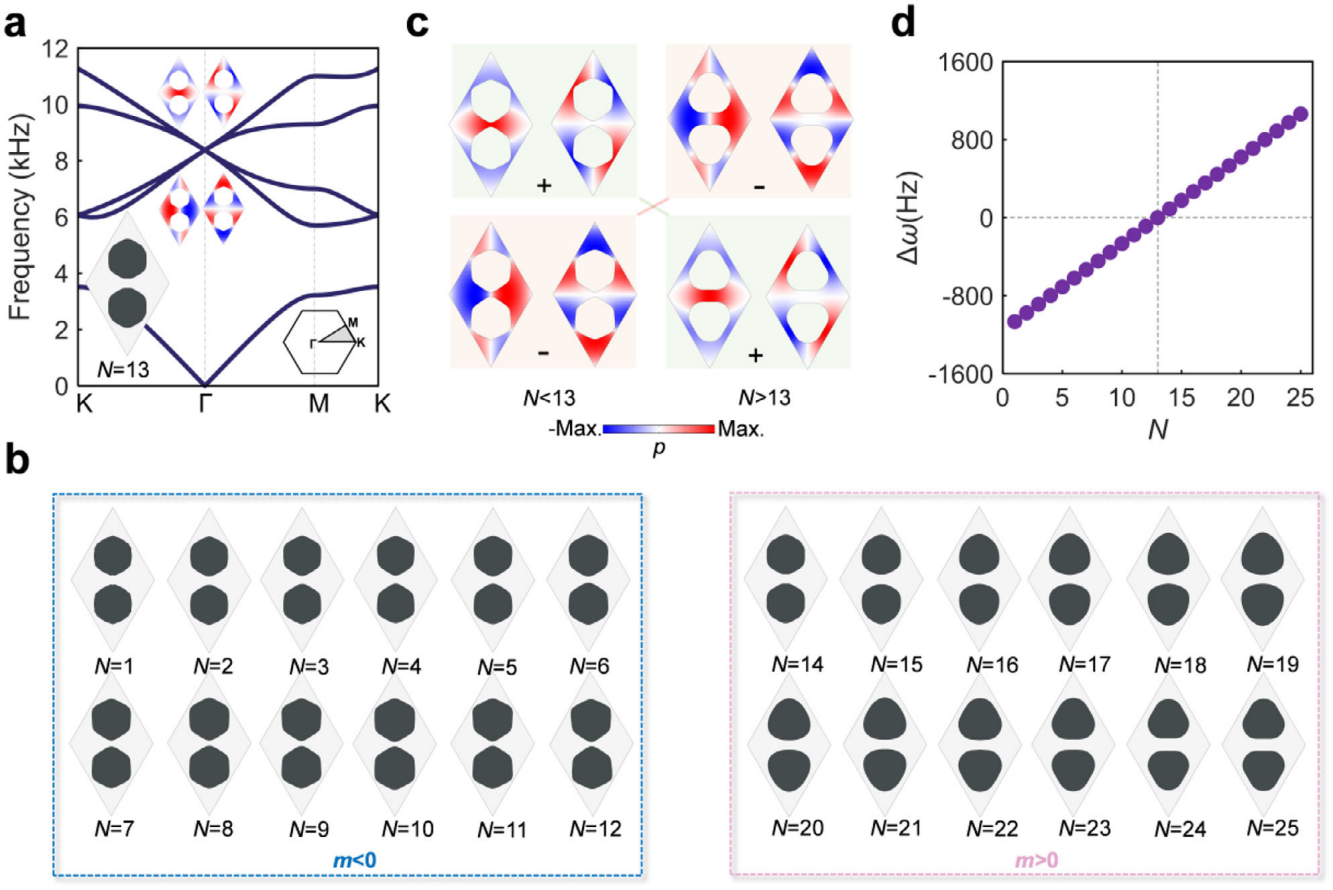
Optimized unit cells with linearly increased effective Dirac masses in the hexagonal lattice. a) The designed unit cell (*N* = 13) with a double Dirac cone and its band diagram. b) The designed unit cells (*N* = 1–12 and *N* = 14–25) with linearly increased local bandgap Δω c) The eigenfields of dipolar and quadrupolar modes for unit cells with *N* < 13 and *N* > 13 at the Г point. d) Values of Δω of the unit cells as a function of *N*.

**Figure 3:**
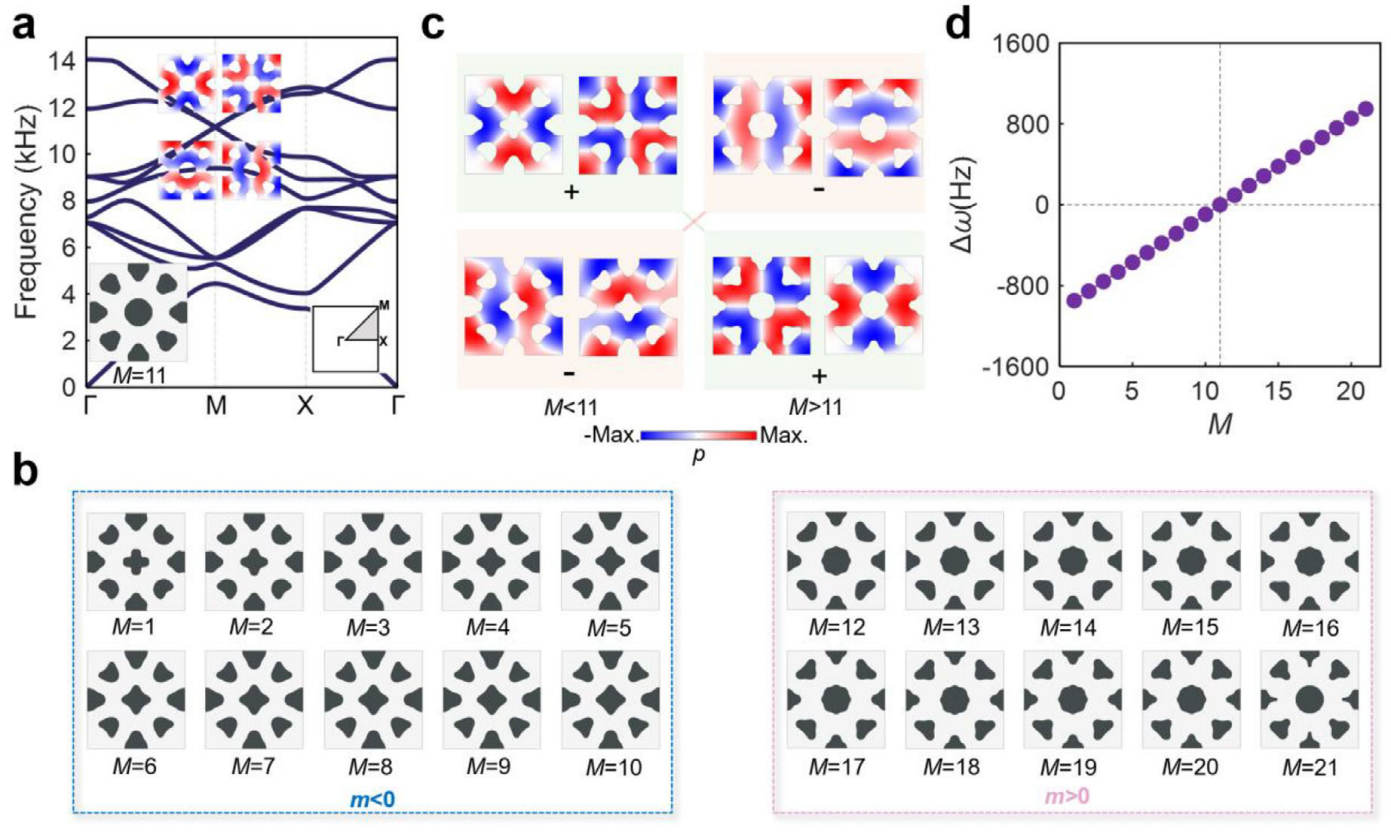
Optimized unit cells with linearly increased effective Dirac masses in the square lattice. a) The designed unit cell *(M* = 11) with a double Dirac cone and its band diagram. b) The designed unit cells (*M* = 1–10 and *M* = 12–21) with linearly increased local bandgap Δω c) The eigenfields of dipolar and quadrupolar modes for unit cells with *M* < 11 and *M* > 11 at the M point. d) Values of Δω of the unit cells as a function of *M*.

We first construct the accidental double Dirac cone degenerated by two dipolar and two quadrupolar modes at the Г point of the hexagonal lattice (as shown in **Figure** **2**a) and the M point of the square lattice (as shown in **Figure** **3**a) with the lattice size of both 4 cm. The unit cell with the double Dirac cone in the hexagonal lattice is designed by the topology optimization method we developed in Section S1 (Supporting Information), which can control the frequencies of the dipolar and quadrupolar modes to be the desirable values.^[^ ^43^ ^]^ We set frequencies of the dipolar and quadrupolar modes at the Г point to be equal to get the double Dirac cone. As our numerical experiences show that, for the square lattice, it is very difficult to achieve four‐fold degeneracy at the Г point, and thus we select the M point to construct the double Dirac cone. The unit cell with the double Dirac cone at the M point in the square lattice is designed using the method in ref. [41]. Meanwhile, we also develop a topology optimization method, as detailed in Section S1 (Supporting Information), to control the frequencies of the dipolar and quadrupolar modes at the M point. The Hamiltonian of the double Dirac cone degenerated by two dipolar and two quadrupolar modes can be described by^[^ ^37^, ^41^ ^]^

(1) $$H=\left(\begin{array}{cc} H_{+} & 0\\ 0 & H_{-} \end{array}\right)$$

with

(2) $$H_{+}=\left(\begin{array}{cc} 0 & v_{D}\left(k_{x}-ik_{y}\right)\\ v_{D}\left(k_{x}+ik_{y}\right) & 0 \end{array}\right)$$

and

(3) $$H_{-}=\left(\begin{array}{cc} 0 & v_{D}\left(k_{x}+ik_{y}\right)\\ v_{D}\left(k_{x}-ik_{y}\right) & 0 \end{array}\right)$$

where *k_x_* and *k_y_* denote the relative wave vectors with respect to the four‐fold degenerate Dirac point along the *x* and *y* directions, and *v_D_* is the Dirac velocity, i.e., the slope of the cone. Note this Hamiltonian describing the four‐fold degenerate Dirac point can be considered as the superposition of two two‐fold‐degenerate Weyl points with opposite chiralities. Then, we set the frequencies of the dipolar and quadrupolar modes with different values to lift the four‐fold degeneracy of the double Dirac cone and open a local bandgap (Δω = ω_*d*_ − ω_*q*_ with ω_*d*_  and ω_*q*_  being frequencies of dipolar and quadrupolar modes respectively), which is equivalent to introducing an effective Dirac mass, *m* = Δω/2, into *H*. As shown in **Figures** 2b and 3b, we design 25 (21) unit cells for the hexagonal (square) lattice, denoted by *N* (*M*), with linearly increased Δω, as per **Figures** 2d and 3d. The detailed frequencies of dipolar and quadrupolar modes for these unit cells are given in Section S2 (Supporting Information). Note that, as illustrated in **Figures** 2c and 3c, for unit cells with *N <* 13 (*M <* 11), the quadrupolar modes are higher in frequency than the dipolar modes with *m <* 0, while they are inverted with *m >* 0 for those with *N >* 13 (*M >* 11). Thus, a linearly increased effective Dirac mass is synthesized. After introducing such a linearly increased mass *m* = γ*y* into the Hamiltonian, *H*_+_ and *H*_−_ can be formulated as

(4) $$H_{+}^{m}=\left(\begin{array}{cc} \gamma y & v_{D}\left(k_{x}-ik_{y}\right)\\ v_{D}\left(k_{x}+ik_{y}\right) & -\gamma y \end{array}\right)$$

(5) $$H_{-}^{m}=\left(\begin{array}{cc} \gamma y & v_{D}\left(k_{x}+ik_{y}\right)\\ v_{D}\left(k_{x}-ik_{y}\right) & -\gamma y \end{array}\right)\;$$

After applying a unitary transformation of $\sigma_{x}\to \sigma_{z},\sigma_{y}\to \sigma_{x},\sigma_{z}\to \sigma_{y}$, we can get

(6) $$H_{+}^{m}=\left(\begin{array}{cc} v_{D}k_{x} & \left(v_{D}k_{y}-i\gamma y\right)\\ \left(v_{D}k_{y}+i\gamma y\right) & -v_{D}k_{x} \end{array}\right)$$

(7) $$H_{-}^{m}=\left(\begin{array}{cc} v_{D}k_{x} & \left(v_{D}k_{y}+i\gamma y\right)\\ \left(v_{D}k_{y}-i\gamma y\right) & -v_{D}k_{x} \end{array}\right)$$

We now define $b=\frac{1}{\sqrt{2\gamma v_{D}}}\left(v_{D}k_{y}-i\gamma y\right)$ and $b^{+}=\frac{1}{\sqrt{2\gamma v_{D}}}\left(v_{D}k_{y}+i\gamma y\right)$, which satisfy [*b*, *b* ^+^] =  1 and then we can get

(8) $$H_{+}^{m}=\left(\begin{array}{cc} v_{D}k_{x} & \sqrt{2\gamma v_{D}}b\\ \sqrt{2\gamma v_{D}}\;b^{+} & -v_{D}k_{x} \end{array}\right)$$

(9) $$H_{-}^{m}=\left(\begin{array}{cc} v_{D}k_{x} & \sqrt{2\gamma v_{D}}b^{+}\\ \sqrt{2\gamma v_{D}}\;b & -v_{D}k_{x} \end{array}\right)$$

The eigenvector of $H_{+}^{m}$ could be written as $\psi_{n}^{+}=\left(\begin{array}{c} \alpha_{n}\left|n-1\right\rangle \\ \beta_{n}\left|n\right\rangle \end{array}\right)$  whereas $H_{-}^{m}$ as $\psi_{n}^{-}=\left(\begin{array}{c} \alpha_{n}\left|n\right\rangle \\ \beta_{n}\left|n-1\right\rangle \end{array}\right)$ . When *n* ≥ 1, the eigenvalues of $H_{+}^{m}$ and of $H_{-}^{m}$ are both $\pm \sqrt{v_{D}^{2}k_{x}^{2}+2n\gamma v_{D}}$. However, when *n*  =  0, $\psi_{0}^{+}=\left(\begin{array}{c} 0\\ \left|0\right\rangle \end{array}\right)$ , which gives $\omega_{0}^{+}=-v_{D}k_{x}$; whereas $\psi_{0}^{-}=\left(\begin{array}{c} \left|0\right\rangle \\ 0 \end{array}\right)$ , which gives $\omega_{0}^{-}=v_{D}k_{x}$. As a result, the zeroth‐order Landau levels of $H=(\begin{array}{cc} H_{+}^{m} & 0\\ 0 & H_{-}^{m} \end{array})$  are ± *v_D_k_x_*, exhibiting the salient feature of spin‐locked helical propagation.

**Figure 4:**
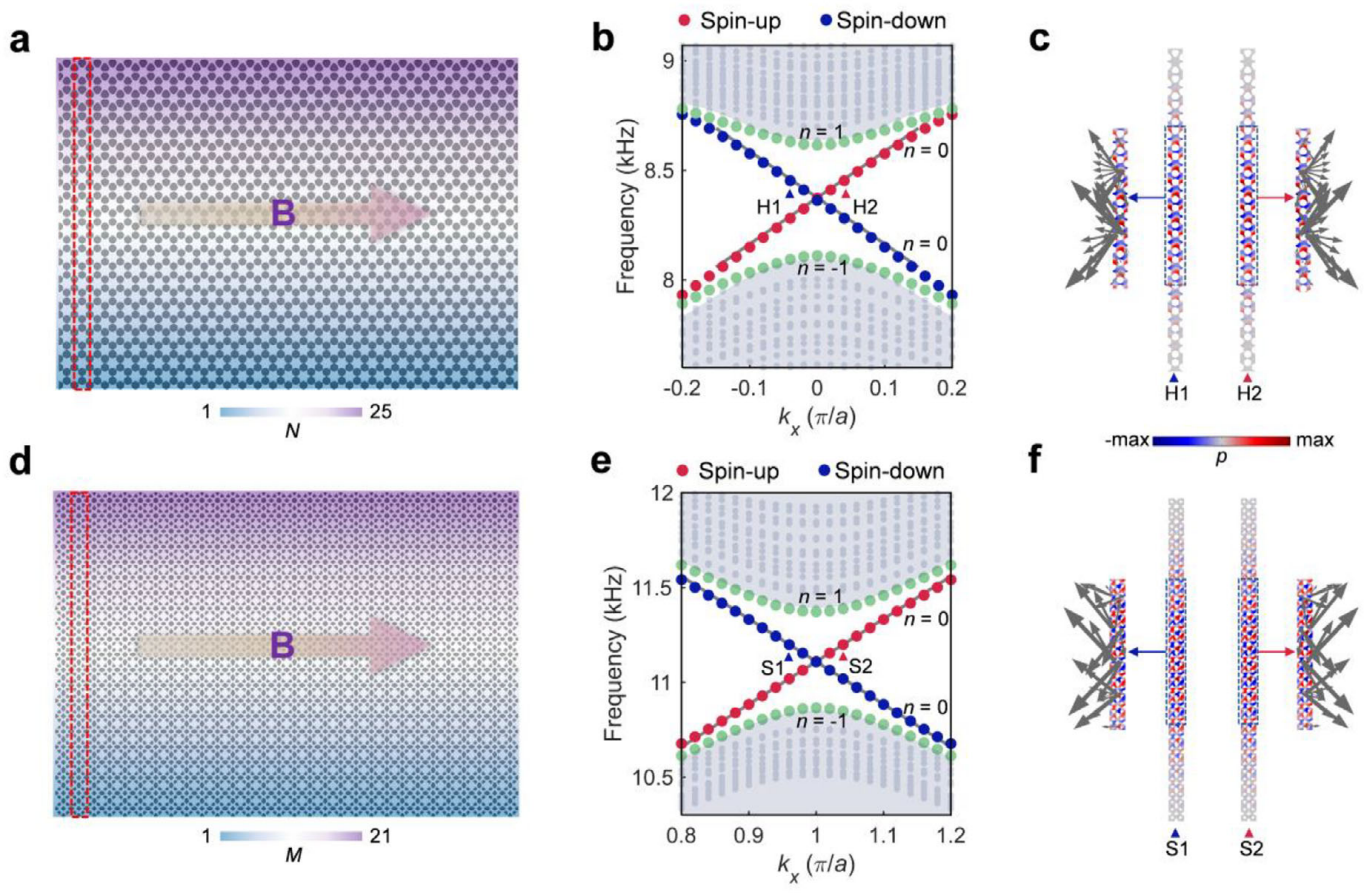
Emergence of helical Landau levels. a,d) Schematic of the supercell made of the designed unit cells with (a) hexagonal and (d) square lattices. b,e) Projected band diagram of the supercell in (a) and (d), respectively. c,f) Eigenfields of the eigenmodes denoted by H1 and H2 in (b) and S1 and S2 in (e), respectively.

**Figure 5:**
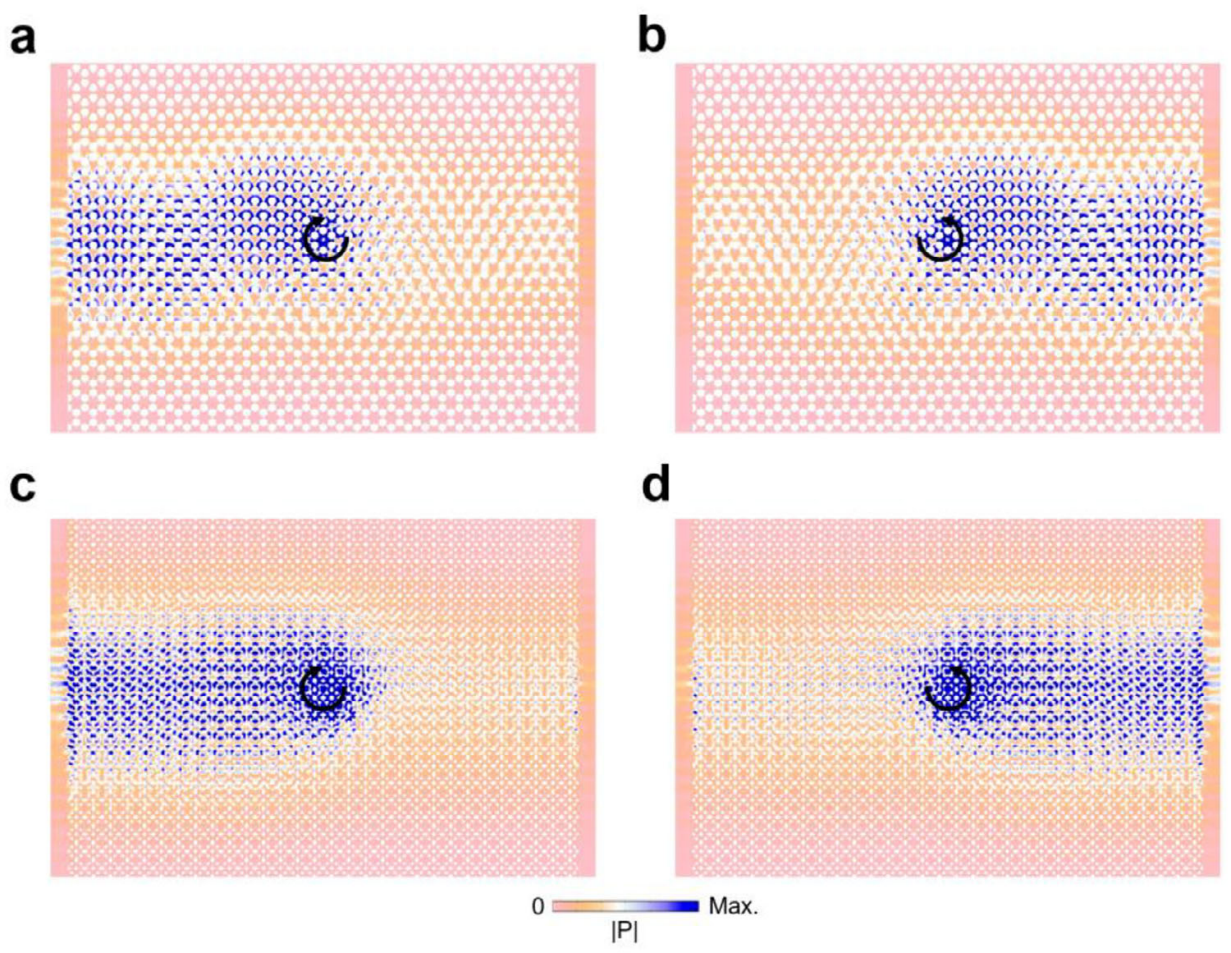
One‐way propagation of the zeroth‐order Landau levels. a) Leftward and b) rightward one‐way propagation of the zeroth‐order Landau levels within the structure with hexagonal lattices. c) Leftward and d) rightward one‐way propagation of the zeroth‐order Landau levels within the structure with square lattices. The arrows denote the rotation direction of the chiral source.

Next, we construct the inhomogeneous supercell using the designed unit cells (as denoted by the red dashed box in **Figure** **4**a,d and then build a structure by arranging the supercell periodically along the *x* direction. In doing so, the in‐plane pseudomagnetic field denoted by the arrow is synthesized. **Figure** 4b,e present the calculated projected band diagram of the supercell in **Figure** 4a,d, respectively. We can find that, for both supercells with both hexagonal and square lattices, zeroth‐order Landau levels represented by a pair of spin‐up and spin‐down states with opposite group velocities emerge, as denoted by the red and blue scatters, respectively, which agree well with the analytical solutions denoted by the solid lines. **Figure** 4c,f show the eigenfields of the eigenmodes denoted by H1 and H2 in **Figure** 4b and **Figure** 4e, respectively. It can be observed that acoustic energies concentrate at the middle domains of the supercell with a large width, providing the opportunity for large‐area acoustic energy transportation. Besides, from the energy flow denoted by the arrows, one can also find that the spin‐up and spin‐down states possess opposite energy flows and thus they can be excited independently using clockwise or counterclockwise chiral sources to achieve unidirectional propagation, as per **Figure** **5**.

**Figure 6:**
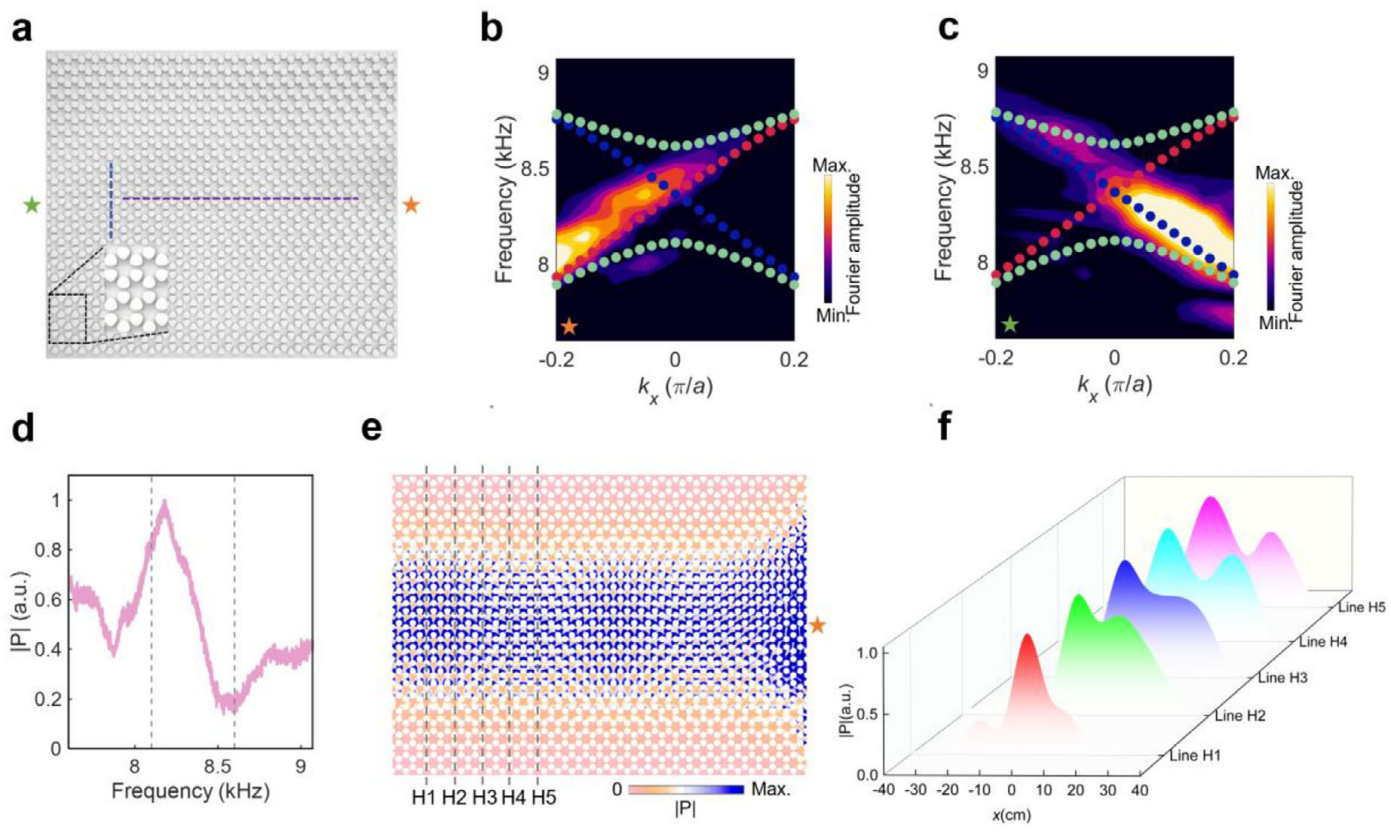
Experimental validation of the spin‐locked helical Landau levels in hexagonal lattices. a) The fabricated sample. b,c) The Fourier transform of the measured acoustic pressure fields along the purple dashed line in (a) when the source is put at (b) right and (c) left ends of the sample, denoted by the orange and green stars, respectively. d) The measured transmission spectra. e) The simulated acoustic fields at 8.1 kHz of the structure. f) The measured acoustic pressure fields along the dashed lines in (e).

**Figure 7:**
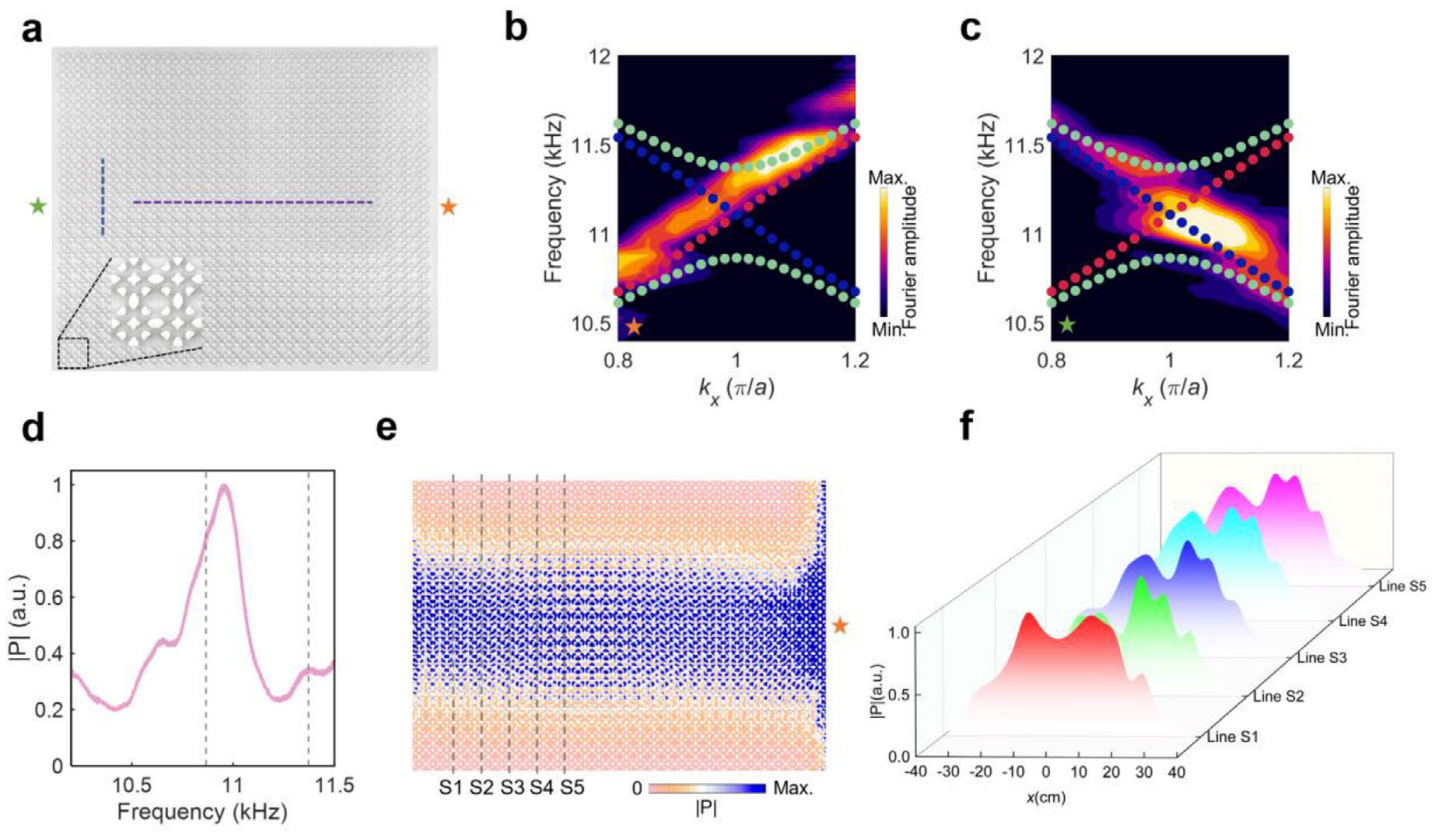
Experimental validation of the spin‐locked helical Landau levels in square lattices. a) The fabricated sample. b,c) The Fourier transform of the measured acoustic pressure fields along the purple dashed line in (a) when the source is put at (b) right and (c) left end of the sample, respectively. d) The measured transmission spectra. e) The simulated acoustic fields at 11 kHz of the structure. f) The measured acoustic pressure fields along the dashed lines in (e).

To validate the spin‐locked helical Landau levels in both hexagonal and square lattices, we fabricate the two structures in **Figure** 4a,d via 3D printing, as presented in **Figures** **6**a and **7**a, respectively. The point source indicated by the star is put at the right (denoted by the orange star) and left (denoted by the green star) ends of the sample for launching acoustic waves to excite spin‐up and spin‐down states, respectively. **Figures** 6b,c and 7b,c present the Fourier transform of the measured acoustic pressure fields along the purple dashed line in **Figures** 6a and 7a when the source is put at the right and left ends of the sample with hexagonal (square) lattice, respectively. We can observe that the spin‐up and spin‐down zeroth‐order Landau levels (denoted by the red and blue circles, respectively) are excited independently and the measured dispersions agree well with the numerical solutions. **Figures** 6d and 7d present the measured transmission spectra, obtained by integrating the absolute pressure along the blue dashed line in **Figures** 6a and 7a when the source is put at the right end of the sample, showing enhanced transmissions within the frequency range between the ±first‐order Landau levels (denoted by the dashed lines). Meanwhile, **Figures** 6e and 7e plot the simulated acoustic fields at 8.1 kHz (11 kHz) of the sample with hexagonal (square) lattice, demonstrating the large‐area transportation of acoustic energies enabled by the zeroth‐order Landau levels. **Figures** 6f and 7f show the measured acoustic pressure fields along the dashed lines in **Figures** 6e and 7e, from which we can observe that energies concentrate within the central region of the sample with a finite width, agreeing well with the simulated fields in **Figures** 6e and 7e.

**Figure 8:**
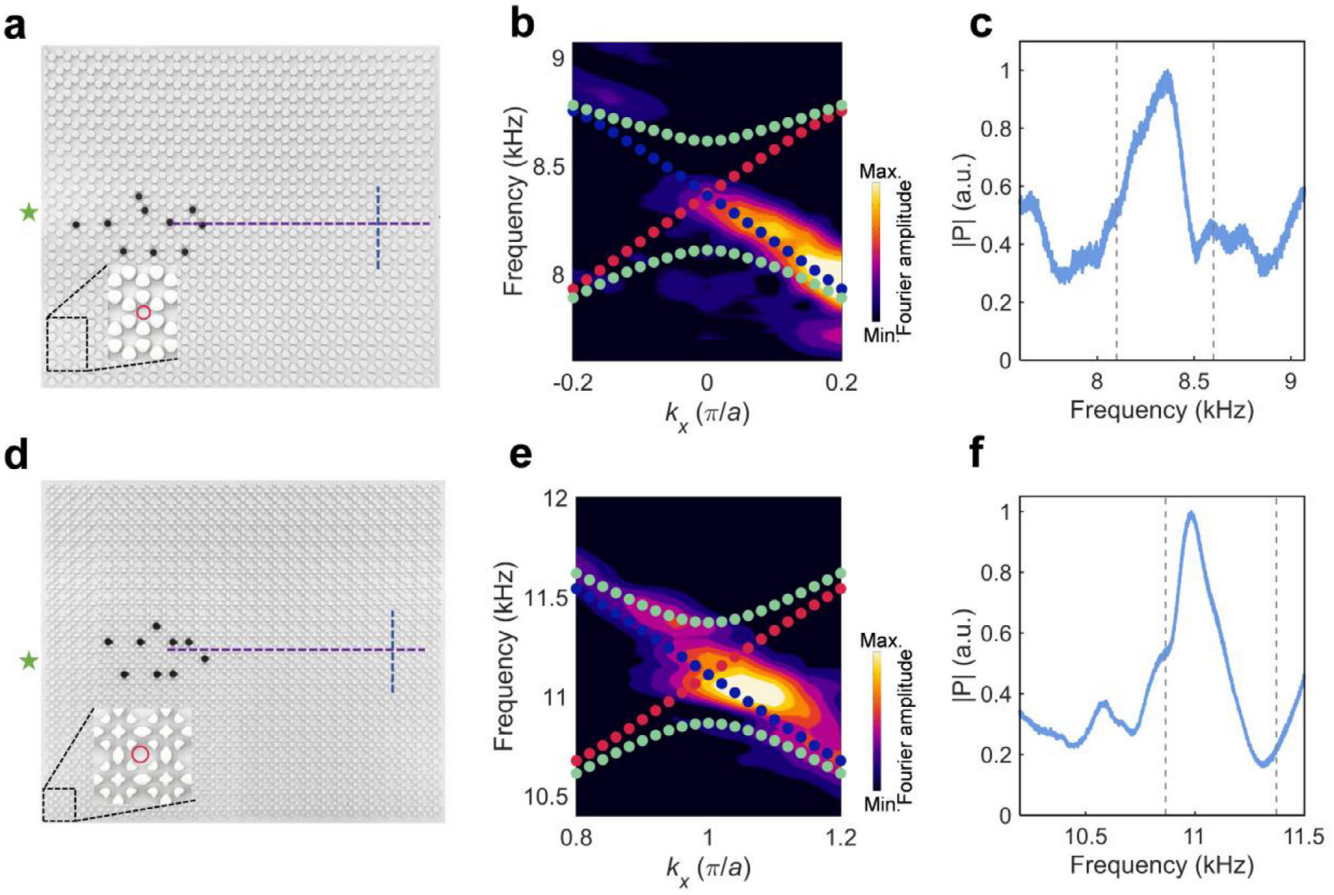
Experimental validation of robustness of the helical Landau levels. a,d) Schematic of the introduced defects within the structure with (a) hexagonal and (d) square lattice. b,e) The Fourier transform of the measured acoustic pressure fields along the purple dashed line in (a) and (d) when the source is put at the left end of the sample. c,f) The measured transmission spectra of the structure with (c) hexagonal and (f) square lattice.

As the zeroth‐order Landau levels have topological origin, they are resilient against defects. To validate this property, we insert several cylinders (denoted by the black color) with a diameter of 2 cm into the samples, as shown in **Figure** **8**a,d, which almost fill the holes denoted by the red circles. **Figure** 8b,e show the measured dispersions of spin‐down zeroth‐order Landau levels, obtained by the Fourier‐transform of the measured acoustic pressure fields along the purple dashed line in **Figure** 8a,d, of the sample with hexagonal and square lattices, respectively, which still agree well with the theoretical solutions after the defects are introduced. Moreover, from the measured transmission spectra shown in **Figure** 8c,f obtained by integrating the absolute pressure along the blue dashed line in **Figure** 8a,d, we can also observe enhanced transmissions within the frequency range between the ±first‐order Landau levels (denoted by the dashed lines), indicating the robustness of the zeroth‐order Landau levels again. Meanwhile, in Section S3 (Supporting Information), we demonstrate that one can achieve flexible large‐area acoustic energy conveying based on Landau levels in square lattices.

## 3 Conclusion

In conclusion, we have successfully realized helical zeroth‐order Landau levels based on spin Hall systems in acoustic crystals with both square and hexagonal lattices. Utilizing the topology optimization method, we design an array of unit cells with linearly increased local bandgaps by lifting the four‐fold degeneracy of the double Dirac cone, which introduces a linearly increased effective mass in the Dirac Hamiltonian, thereby synthesizing in‐plane pseudomagnetic fields. The emergence of spin‐locked helical Landau levels has been confirmed, and their dispersions have been experimentally measured. We demonstrate the large‐area conveyance of acoustic energy facilitated by these helical Landau levels, as well as their robustness against defects. Our work paves the way for exploring topological zeroth‐order Landau levels through the framework of spin Hall physics. Furthermore, the developed design methodology can also be extended to engineer helical Landau levels in photonic and elastic phononic crystals, as well as in 3D classical wave systems. Moreover, this methodology can be exploited to simultaneously construct synthetic electric and magnetic fields to further achieve rainbow Landau levels in photonic and phononic systems.^[^ ^44^ ^]^

## 4 Experimental Section

The specimen was fabricated using 3D printing with photosensitive resin, with a unit cell thickness of 10 mm. A chirp signal was generated by a Sound and Vibration Module (PXIe‐4464) to drive the sound source. The desired point source was created using a balanced armature unit (Bellsing 30095). A ¼‐inch microphone was used to measure the acoustic pressure field. The measured signal was amplified by a signal conditioner (BK1704) and subsequently acquired by the sound and vibration module. Data processing, including the Fast Fourier‐Transform (FFT), was performed on a host PC running LabVIEW, which communicated with the Sound and Vibration Module (PXIe‐4464). For spatial measurements, a sliding strip holding the microphone moved stepwise along the *x*‐direction, while the movement of the cover in the *y*‐direction enabled the switching of the scanned lines. The schematic of the experimental devices is given in Section S4 (Supporting Information).

## Conflict of Interest

The authors declare no conflict of interest.

## Supporting information



Supporting Information

## Data Availability

The data that support the findings of this study are available from the corresponding author upon reasonable request.
