# Supplementary material for: Realization of Spin‐locked Acoustic Helical Landau Levels in both Hexagonal and Square Lattices
Source: Adv Sci (Weinh). 2025 Jun 27;12(36):e07059. doi: 10.1002/advs.202507059 (PMC12462949; doi:10.1002/advs.202507059)
Supplement: Supplementary file 1 — Supporting Information [file ADVS-12-e07059-s001.docx]

# Supplementary Materials for “Realization of spin-locked acoustic helical Landau levels in both hexagonal and square lattices”

Yafeng Chen^1^, Zhihao Lan^2^, Shanjun Liang^3⸷^, Lei Fan^4^, Jie Zhu^1⸾^, and Zhongqing Su4[[1]](#footnote-1)*

^1^*Institute of Acoustics, School of Physics Science and Engineering, Tongji University, 200092 Shanghai, China*

^2^*Department of Electronic and Electrical Engineering, University College London, London WC1E 7JE, United Kingdom*

^3^*Division of Science, Engineering and Health Studies, College of Professional and Continuing Education, Hong Kong Polytechnic University, Hong Kong SAR, China*

^4^*Department of Mechanical Engineering, The Hong Kong Polytechnic University, Kowloon, Hong Kong SAR, China*

# 1. The topology optimization method for engineering acoustic Landau levels

**1.1. Square lattice**

When there is no source, the eigenvalue equation of acoustic crystals is [R1]

$\text{-}\nabla\cdot\left(\frac{1}{\rho\left(r\right)}\nabla p\left(r,k\right)\right)+\frac{1}{B\left(r\right)}\frac{\partial^{2}p\left(r,k\right)}{\partial t^{2}}=0$ (S1)

where $B$ and $\rho$ denote bulk modulus and mass density, respectively; $\rho\left(r\right)=\rho\left(r+R\right)$ and $\rho\left(r\right)=\rho\left(r+R\right)$, with **R** and $r\left(x,y\right)$ representing the lattice translation vector and position vector, respectively. Following the Bloch-Floquet theory [R2, R3], $p\left(r,k\right)$ can be formulated as $p_{k}\left(r,k\right)e^{i\left(\omega t+k\cdot r\right)}$, where $p_{k}\left(r,k\right)=p_{k}\left(r+R,k\right)$, with *ω*, $k=\left(k_{x},k_{y}\right)$, $p_{k}\left(r\right)$ denoting the angular frequency, Bloch wave vector, and periodic function, respectively. Based on the finite element theory, Eq. (S1) can be expressed by

$\left(K\left(k\right)-\omega^{2}M\right)P=0$ (S2)

where **P**, **K**, **M** represent the nodal pressure vector, global stiffness matrix and global mass matrix, respectively. We can get the band diagram of the acoustic crystals by sweeping the wave vector along the boundary of the first irreducible Brillouin zone and solving Eq. (S2).

In the main text, the unit cells with square lattice denoted by *M* = 1-21 are designed with linearly increased local bandgap $\Delta\omega$ ($\Delta\omega\text{=}\omega_{d}-\omega_{q}$), respectively. For designing each unit cell, $\omega_{d}$ and $\omega_{q}$ are optimized to be the specific values $\omega_{1}$ and $\omega_{2}$. Here, we select the trivial and nontrivial unit cells in Ref. [R4] with C_4v_ symmetry as the initial structures for designing the unit cells with *M* <11 and *M* >11, respectively. For the initial unit cells, the eigenmodes of the 6*^th^*-9*^th^* bands at M point are two dipolar and two quadrupolar modes that we focus on. Meanwhile, we need to ensure that two dipolar (quadrupolar) modes, with their frequencies denoted by $\omega_{d1}$ and $\omega_{d2}$ ($\omega_{q1}$ and $\omega_{q2}$), are degenerated. Thus, the optimization objective is set to minimize the following equation

$g=\left(\omega_{d1}-\omega_{1}\right)^{2}+\left(\omega_{d2}-\omega_{1}\right)^{2}+\left(\omega_{q1}-\omega_{2}\right)^{2}+\left(\omega_{d2}-\omega_{2}\right)^{2}$ (S3)

which is equal to maximizing $f=-g$. Then, the sensitivity of the objective function about the design variable $x_{e}$ can be formulated as

$\frac{\partial f}{\partial x_{e}}=-2\left[\left(\omega_{d1}-\omega_{1}\right)\frac{\partial\omega_{d1}}{\partial x_{e}}+\left(\omega_{d2}-\omega_{1}\right)\frac{\partial\omega_{d2}}{\partial x_{e}}+\left(\omega_{q1}-\omega_{2}\right)\frac{\partial\omega_{q1}}{\partial x_{e}}+\left(\omega_{q2}-\omega_{2}\right)\frac{\partial\omega_{q2}}{\partial x_{e}}\right]$ (S4)

where $\frac{\partial\omega_{t}}{\partial x_{e}}$ (*t* = *d*1, *d*2, *q*1 and *q*2) can be derived by differentiating both sides of Eq. (S2):

$\frac{\partial\omega_{t}}{\partial x_{e}}\text{=}\frac{1}{2\omega_{t}}P^{T}\left(\frac{\partial K}{\partial x_{e}}-\omega^{2}\frac{\partial M}{\partial x_{e}}\right)P$ (S5)

Upon calculating the sensitivity of the objective function about all elements, we adopt the Bi-directional evolutionary structural optimization (BESO) method to update the design variables iteratively to maximize the objective function [R5]. Once the objective function is maximized to be 0 approximatively, two dipolar modes and two quadrupolar modes are degenerated at the specific frequencies $\omega_{1}$ and $\omega_{2}$, respectively. By elaborately setting $\omega_{1}$ and $\omega_{2}$, we can get the unit cells with linearly increased $\Delta\omega$, as presented in the main text. Meanwhile, during the optimization, the unit cell is constrained with C_4v_ symmetry. For a structure with C_4v_ symmetry, it possesses four-fold rotational symmetry and four reflection symmetries, simultaneously. Here, we discretize the unit cell with 64×64 elements and assign each element with a design variable and then use a matrix, named *rho*, with dimensions of 64×64 to store these design variables. After updating design variables at each iteration, we use the command “*rho* = (*rho* + *rho’*+ rot90(*rho*, 1) + rot90(*rho*, 1)’ + rot90(*rho*, 2) + rot90(*rho*, 2)’ + rot90(*rho*, 3) + rot90(*rho*, 3)’)/8” in Matlab to impose four-fold rotational symmetry and four reflection symmetries simultaneously.

**1.2. Hexagonal lattice**

For acoustic crystals in hexagonal lattice with C_6v_ symmetry, two dipolar modes or quadrupolar modes are determinedly degenerate at the Г point. The dipolar or quadrupolar modes can be excited at specific frequencies by simultaneously maximizing the power emitted by a harmonic sound source at the specific position [R6], as sketched in **Figure S1**. Here, the source of $iJ\left(r\right)e^{i\omega t}$ is selected and the governing equation under such excitation is

$\left(K-\omega^{2}M\right)P=iJ$ (S6)

Then, the power emitted by the assigned source can be calculated by

$f\left(\omega,J\right)=\text{-}\mathrm{Re}\left(J^{\text{*}}\cdot P\right)$ (S7)

where $J^{*}$ is the complex conjugate of $J$. Here, to avoid the ill-solution and ensure the convergence of the optimization, we select the frequency-averaged power to excite the specific modes, defined as

$\tilde{f}\left(\omega,J\right)=f\left(\omega+i\psi,J\right)$ (S8)

Thus, the frequency integral can be replaced by a single evaluation of *f* at a complex frequency $\tilde{\omega}=\omega+i\psi$ [R7]. $\psi$ is set as 0.02 in this paper.


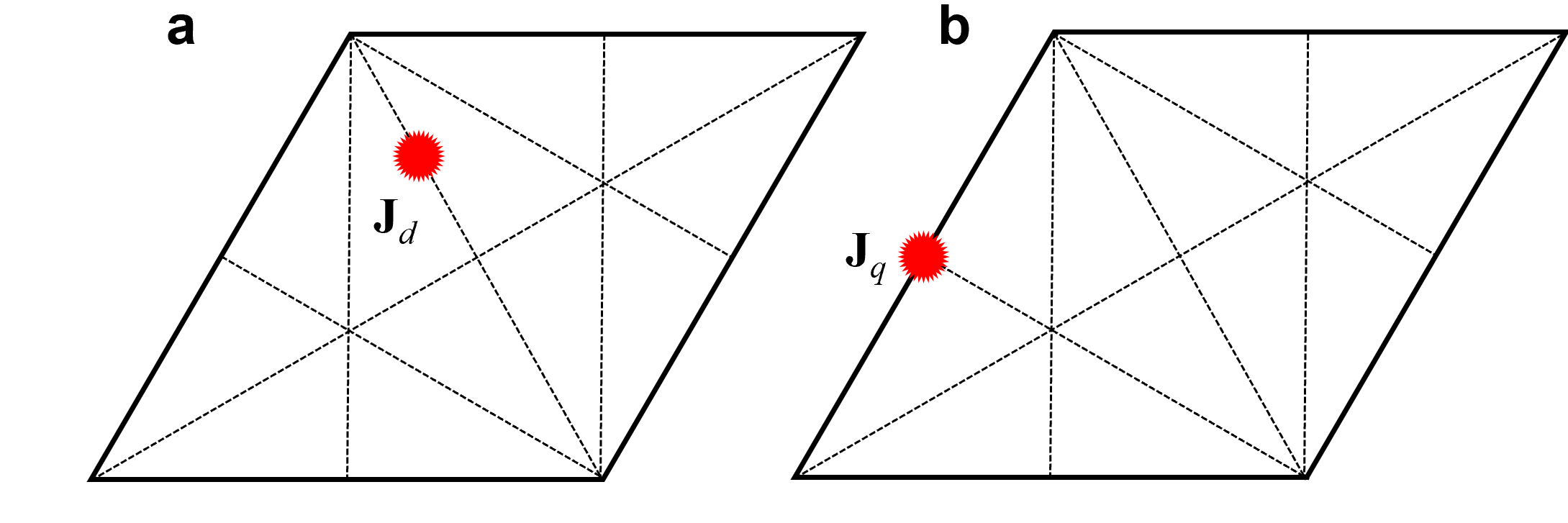


**Figure S1**. Schematic diagram for the positions of the point sources to obtain the desired modes: (a) **J***_d_* for dipolar mode; (b) **J***_q_* for quadrupolar mode.

For designing the unit cells denoted by N=1-25 in the main text, we should trigger dipolar modes and quadrupolar modes at specific $\omega_{d}$ and $\omega_{q}$, which needs to maximize $\tilde{f}_{d}\left(\omega_{d},J_{d}\right)$ and $\tilde{f}_{q}\left(\omega_{q},J_{q}\right)$ simultaneously. For simplicity, $\tilde{f}_{d}\left(\omega_{d},J_{d}\right)$ and $\tilde{f}_{q}\left(\omega_{q},J_{q}\right)$ are replaced by $\tilde{f}_{\text{d}}$ and $\tilde{f}_{\text{q}}$ hereafter. This problem can be equivalently expressed by minimizing a larger one among the inverses of $\tilde{f}_{\text{d}}$ and $\tilde{f}_{\text{q}}$. Such a typical min-max optimization problem is usually non-differentiable and the problem is thus recast in the so-called bound formulation [R8] as

$\left\{\begin{matrix} \text{Find}: x_{e} (e=1, 2, \cdots, N) \\ min:t \\ \text{s.t.} : \left(K-\tilde{\omega}_{\text{d}}^{2}M\right) p_{\text{d}}=iJ_{\text{d}}; \left(K-\tilde{\omega}_{\text{q}}^{2}M\right) p_{\text{q}}=iJ_{\text{q}} \\ 1/\tilde{f}_{\text{d}}(\omega_{\text{d}}, \mathbf{J}_{\text{d}})\leq t; 1/\tilde{f}_{\text{q}}(\omega_{\text{q}}, \mathbf{J}_{\text{q}})\leq t \\ x_{e}\in\left[0,1\right] \end{matrix}\right.$ (S9)

where *t* is the objective function, which selects the larger one among the inverses of $\tilde{f}_{\text{d}}$ and $\tilde{f}_{\text{q}}$. Meanwhile, it also acts as the upper bound of the inverses of $\tilde{f}_{\text{d}}$ and $\tilde{f}_{\text{q}}$. $x_{e}$ (*e* = 1, 2, ...) is the design variable. $x_{e}$=1 means that the element is full of material 2, e.g., polyethylene, while $x_{e}$= 0 denotes material 1, e.g., air. Then, we adopt the method of moving asymptotes (MMA) to update the design variable [R8, R9]. The detailed optimization process can be also referred to Ref. [6]. Meanwhile, we use the following Matlab code to impose C_6v_ symmetry to the unit cell, where *rho* is a 96×96 matrix storing the design variables and ne is set to be 96 herein.

function [rho] = symme(rho,ne)

temp1 = tril(df);

for ind2 = 1:ne

for ind1 = ind2:ne

temp2(ind1,ind2) = temp1(nx-ind1+ind2,ind2);

end

end

for ind1 = 1:ne

for ind2 = 1:ind1

temp3(ind1,ind2) = temp1(ind1,ind1-ind2+1);

end

end

temp4 = (temp1 + temp2 + temp3 + rot90(temp1,2)' + rot90(temp2,2)' + rot90(temp3,2)')/6;

df = temp4 + temp4'-diag(diag(temp4));

end

# 2. The Frequency range of the bandgap for the optimized unit cells

**Figure S2a** and **S2b** present frequency range of the bandgap for the optimized unit cells with square lattice and hexagonal lattice, respectively, where the blue and purple points denote the frequencies of quadrupolar and dipolar modes, respectively, and the black points denote the central frequency of the bandgap. We can observe that the central frequency of the bandgap for the optimized unit cells keep almost the same.


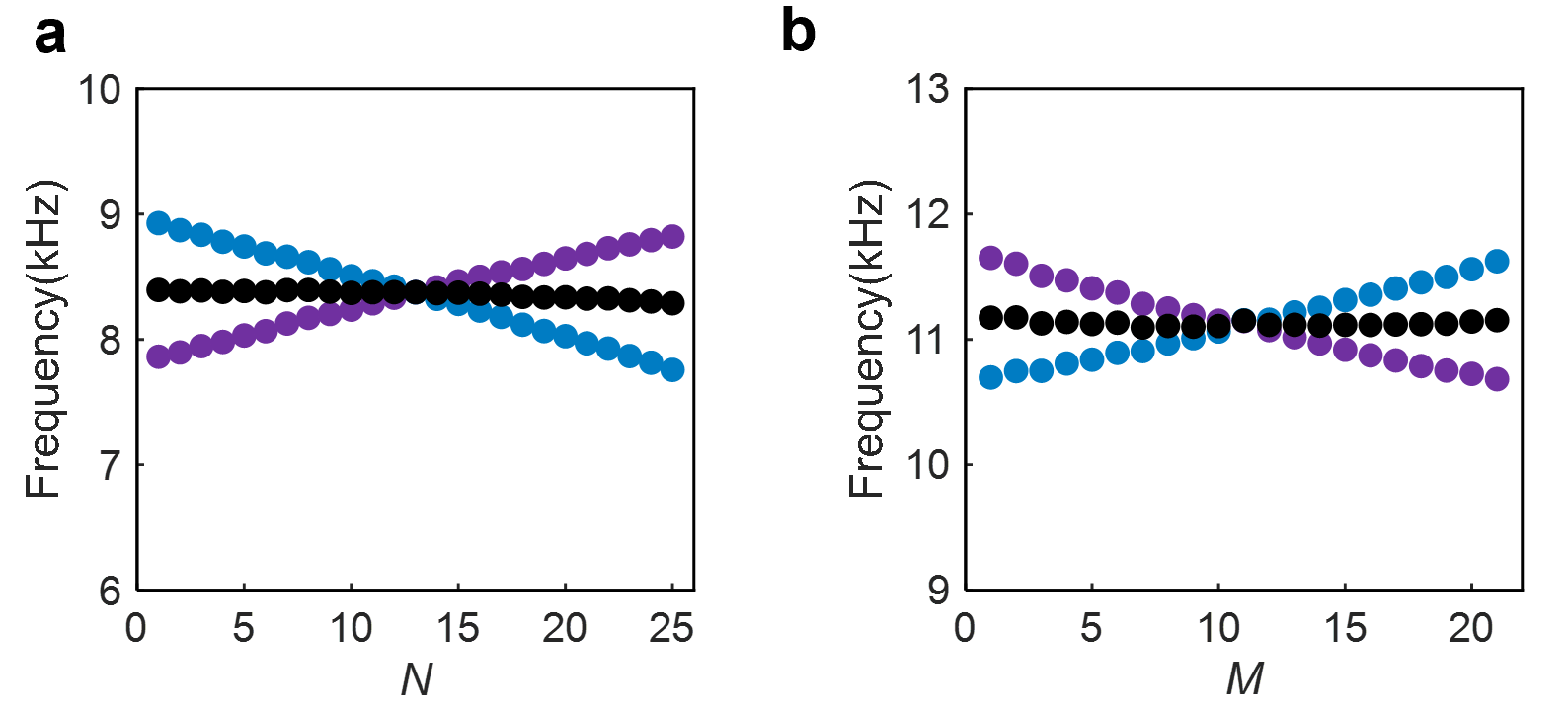


**Figure S2**. Frequency range of the bandgap for the optimized unit cells with (a) square lattice and (b) hexagonal lattice. The blue and purple points denote the frequencies of quadrupolar and dipolar modes, respectively; the black points denote the central frequency of the bandgap.

# 3. Flexible large-area acoustic energy conveying based on the Landau levels in square lattices

Based on Landau levels in the square lattice, we can achieve flexible large-area acoustic energy conveying. As sketched in **Figure S3a,** we customize a curved propagation path (denoted by the blue line) by shifting the specific block. **Figure S3b** shows the absolute pressure fields within the structure under the frequency of 11kHz, demonstrating the flexible large-area acoustic energy conveying along the customized route.


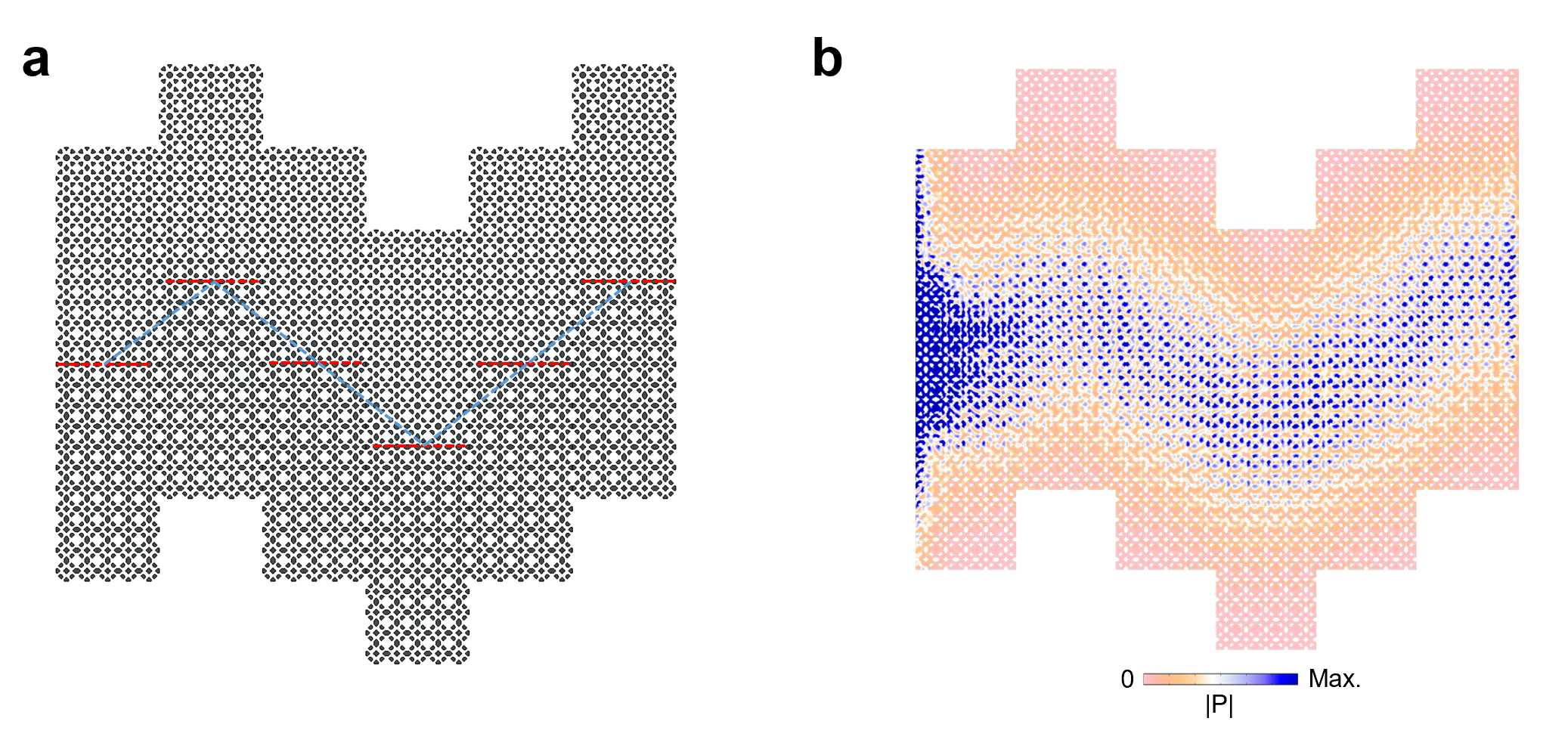


**Figure S3**. Demonstration of flexible large-area acoustic energy conveying based on the Landau levels in square lattices. (a) Schematic of the structure with a curved propagation path. (b) Absolute pressure fields within the structure.

# 4. The experimental details

The schematic of the experimental devices is given in **Figure S4**. The specimen was fabricated using 3D printing with photosensitive resin, with a unit cell thickness of 10 mm. A chirp signal was generated by a Sound and Vibration Module (PXIe-4464) to drive the sound source. The desired point source was created using a balanced armature unit (Bellsing 30095). A ¼-inch microphone was used to measure the acoustic pressure field. The measured signal was amplified by a signal conditioner (BK1704) and subsequently acquired by the sound and vibration module. Data processing, including the Fast Fourier Transform (FFT), was performed on a host PC running LabVIEW, which communicated with the Sound and Vibration Module (PXIe-4464). For spatial measurements, a sliding strip holding the microphone moved stepwise along the *x*-direction, while movement of the cover in the y-direction enabled switching of the scanned lines.


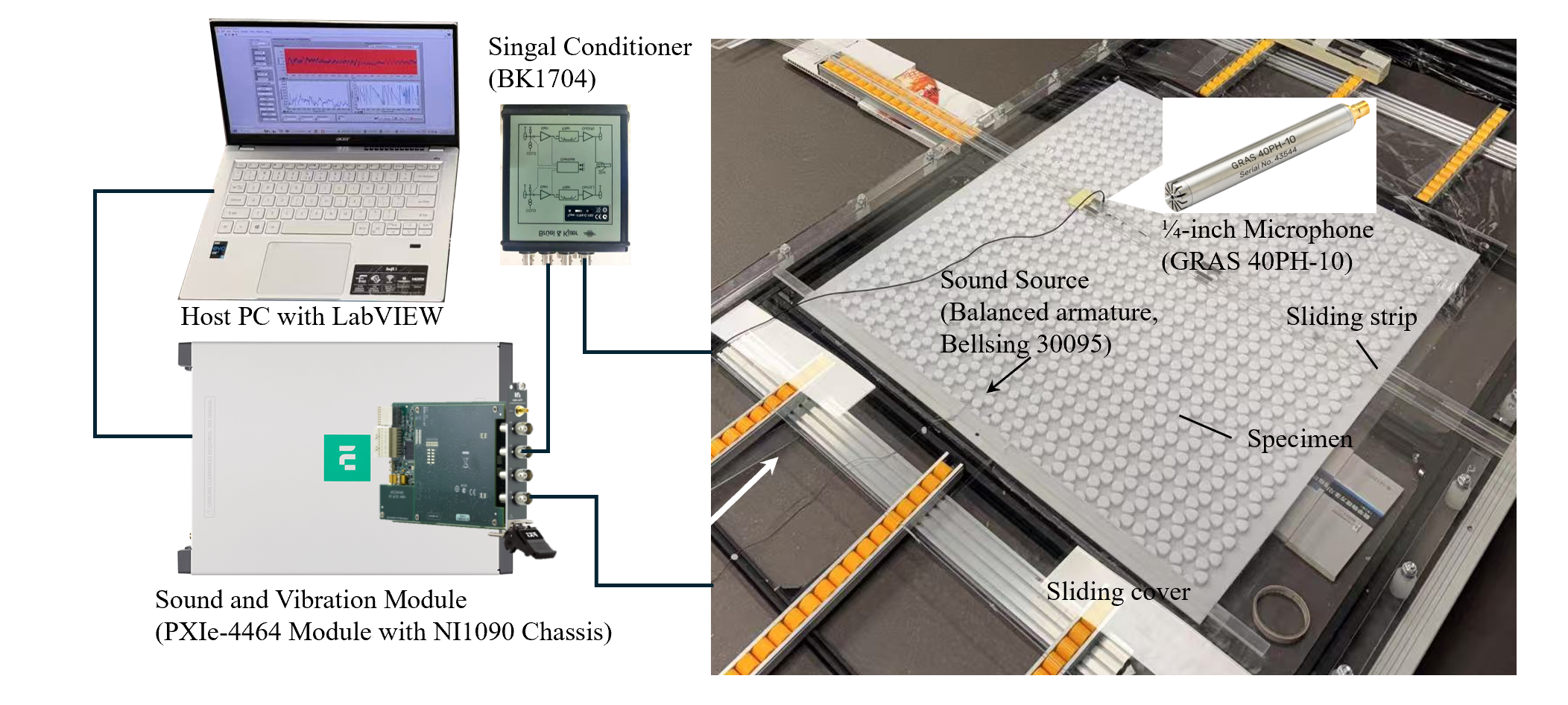


**Figure S4**. Schematic of the experimental devices

**Reference:**

[R1] Laude V. Phononic crystals: Artificial crystals for sonic, acoustic, and elastic waves. Walter de Gruyter GmbH & Co KG, 2015

[R2] Kittel C. Introduction to solid state physics : 6th ed. American Journal of Physics, 2011, 61: 59

[R3] Wiltshaw R, Craster RV, Makwana MP. Asymptotic approximations for bloch waves and topological mode steering in a planar array of neumann scatterers. Wave Motion, 2020, 99: 102662

[R4] Lu Y, Park HS. Double dirac cones and topologically nontrivial phonons for continuous square symmetric c 4 (v) and c 2 (v) unit cells. Physical Review B, 2021, 103: 064308

[R5] Huang X, Xie Y. Evolutionary topology optimization of continuum structures: Methods and applications. John Wiley & Sons, 2010

[R6] Chen Y, Meng F, Huang X. Creating acoustic topological insulators through topology optimization. Mechanical Systems Signal Processing, 2021, 146: 107054

[R7] Liang X, Johnson SG. Formulation for scalable optimization of microcavities via the frequency-averaged local density of states. Optics Express, 2013, 21: 30812-30841

[R8] Svanberg K. A class of globally convergent optimization methods based on conservative convex separable approximations. Society for Industrial and Applied Mathematics, 2002

[R9] Nocedal J, Wright SJ. Numerical optimization. Springer, 1999

1. Corresponding authors:

   ^⸷^junot.liang@cpce-polyu.edu.hk

   ^⸾^ [jiezhu@tongji.edu.cn](mailto:jiezhu@tongji.edu.cn)

   ^*^ [zhongqing.su@polyu.edu.hk](mailto:zhongqing.su@polyu.edu.hk) [↑](#footnote-ref-1)
